# Screening and Brief Intervention in the Criminal Justice System

**Published:** 2004

**Authors:** Sandra Lapham

**Affiliations:** Sandra Lapham, M.D., M.P.H., is director of the Behavioral Health Research Center of the Southwest in Albuquerque, New Mexico

**Keywords:** offender, prison inmate, correctional system and facility, parolee, criminality, recidivism, violence, alcohol use disorders, alcohol use and dependence, intoxication, drinking and driving, impaired drivers, comorbidity, identification and screening, self-report, brief intervention, interview, motivational interviewing, treatment, barriers to treatment, prison-based prevention, literature review

## Abstract

A large proportion of offenders in the criminal justice system have alcohol-related problems. Therefore, it makes sense to implement alcohol screening and brief intervention programs for people in this setting, particularly for impaired driving offenders, who are likely to be alcohol dependent. Although most States mandate screening for impaired drivers, not much effort has been put forth to determine how the screening process could be improved and expanded to the entire criminal justice population. For example, more research is needed on the potential therapeutic benefit of the screening process and on how brief motivational interventions could be incorporated into this process to improve outcomes. To address this, more emphasis should be placed on developing and implementing national standards for screening programs in the criminal justice system, evaluating existing programs, and assuring that these programs provide adequate treatment services to offenders.

Alcohol misuse^1^ not only is linked to multiple health problems but also increases the potential for violent and criminal behavior. In fact, criminal activity is more closely linked to use of alcohol than to any other drug. For example, the 2002 National Crime Victimization Survey found that 21.6 percent of victims of violent crimes thought or knew the offender involved had consumed alcohol alone or together with other drugs, and an additional 1.5 percent of victims thought the offender had used either alcohol or other drugs (AODs) (Bureau of Justice Statistics 2003). Other analyses found that about 40 percent of fatal motor vehicle crashes involved alcohol use (Bureau of Justice Statistics 1998). Moreover, about 40 percent of offenders on probation, in State prisons, or in local jails reported that they had been using alcohol at the time of their offense (Bureau of Justice Statistics 1998). The impact of alcohol use on U.S. crime rates is further emphasized by the large number of people arrested annually for driving while impaired (DWI). In 2001, there were 1.4 million DWI arrests, making this the number one crime, besides drug possession, for which Americans are arrested (NHTSA 2003).

These observations indicate that a large number of people in the criminal justice system (i.e., inmates and probationers) have alcohol-use problems that should be addressed to prevent recidivism. This article describes the scope of alcohol problems among criminal justice populations, summarizes current knowledge about alcohol screening programs with these populations, and reviews the existing literature on the usefulness of these programs. Brief intervention approaches used in the criminal justice system also are discussed. The article concludes with recommendations for improving the alcoholism treatment services offered to clients in the criminal justice system.

## How Does Alcohol Use Contribute to Criminal Behavior?

### Physiological Factors

The association between alcohol use and criminal behavior is based at least in part on alcohol’s diverse physiological effects. In lower amounts, alcohol has a stimulating effect, acting both directly and indirectly on the brain’s pleasure center to induce a “high” that may motivate the drinker to consume more alcohol. As the person drinks more, however, alcohol begins to cause sedative and toxic effects, such as problems coordinating movements, longer reaction times, neurocognitive impairment (i.e., impaired judgment, attention problems, and mood changes), and perceptual distortion. In drinking drivers, impaired coordination and longer reaction times can contribute to traffic crashes; in other situations, cognitive impairment or perceptual distortions can increase the risk of other violent behaviors.

Aggressive and impulsive behavior is more frequently associated with the stimulatory effects of alcohol. Moreover, alcohol intoxication is more likely to stimulate violent actions in people predisposed to aggressive behavior, such as those who are highly impulsive or who have antisocial personality disorder (Moeller et al. 1998). Thus, alcohol use works through a variety of physiological mechanisms to increase a person’s likelihood of perpetrating crimes. Consistent with this observation, epidemiological studies have demonstrated that reduced alcohol consumption in a population is associated with a decline in the rate of violent crimes (Greenfield and Henneberg 2001).

**At a Glance**

**The Scope of Alcohol Problems in the Criminal Justice System**

- 21.6 percent of victims of violent crimes thought or knew the offender had consumed alcohol; another 1.5 percent of the victims thought the offender had used either alcohol or another drug (Bureau of Justice Statistics 2003).
- 40 percent of offenders on probation, in State prisons, or in local jails reported using alcohol at the time of their offense (Bureau of Statistics 1998).
- There were 1.4 million DWI arrests in 2001, making DWI the number one crime, besides drug possession, for which Americans are arrested (NHTSA 2003).
- 18 percent of Federal prison inmates and about 25 percent of State prison inmates reported having experienced problems consistent with a history of alcohol abuse and dependence (Knight et al. 2002).
- 29 percent of Federal and 40 percent of State prisoners reported a previous domestic violence dispute involving alcohol (Knight et al. 2002).
- About two-thirds of convicted DWI offenders are alcohol dependent (Lapham et al. 2001).
- At least one-fourth of AOD-dependent offenders have lifetime histories of major depression or some form of bipolar disorder; 44 percent of inmates in a metropolitan jail had lifetime substance abuse disorders co-occurring with either depression or antisocial personality disorder (Vigdal et al. 1995).

In a study of first-time DWI offenders interviewed 5 years after first being referred to screening following their DWI offense (Lapham et al. 2001):

- 85 percent of female and 91 percent of male DWI offenders had met the criteria for alcohol abuse or dependence at some time in their lives.
- 32 percent of female and 38 percent of male offenders had met criteria for abuse of or dependence on another drug at some time in their lives.
- 50 percent of women with an alcohol use disorder and 33 percent of men with an alcohol use disorder also had at least one psychiatric disorder (not drug-related), most commonly depression and post-traumatic stress disorder.

### Environmental Influences

Because increased alcohol availability is associated with higher alcohol consumption, communities where alcohol is readily available experience higher rates of alcohol-related crimes. Studies analyzing the effects of alcohol availability typically find that when access to alcohol is limited or inconvenient, or when alcohol is more expensive to purchase, the prevalence of alcohol-related problems (e.g., the number of traffic crashes and alcohol-related deaths) is reduced (Edwards et al. 1994; Gruenewald et al. 2000). These observations suggest that implementing environmental control strategies to limit alcohol use (e.g., by restricting advertising, sales, and distribution of alcohol, or by increasing taxes on alcohol) and strictly enforcing laws against sales to minors or intoxicated people as well as laws against driving after drinking are promising approaches to reducing alcohol-related crime.

## Epidemiology of Alcohol Misuse in Criminal Justice Populations

### Scope of Alcohol Problems

By the end of 2003, about 1.47 million people were incarcerated in U.S. Federal and State prisons, and an additional 4.85 million were on probation or parole (Bureau of Justice Statistics 2004a,b). Many of these people either reported alcohol use problems or were involved with the criminal justice system as a direct result of alcohol misuse (e.g., people convicted of DWI offenses). About 18 percent of Federal prison inmates and about 25 percent of State prison inmates reported having experienced problems consistent with a history of alcohol abuse or dependence (Knight et al. 2002). Alcohol misuse plays a particularly large role in domestic violence and DWI offenses: 29 percent of Federal and 40 percent of State prisoners report having been involved in a domestic violence dispute involving alcohol (Knight et al. 2002). These statistics demonstrate a need to routinely screen all offenders in the criminal justice system for alcohol misuse.

### Scope of Criminal Justice Referrals to Treatment

No accurate statistics on the total number of criminal justice referrals to community-based alcoholism treatment programs are available. These referrals are numerous, however, constituting an estimated 40 to 50 percent of referrals to community-based programs (Anglin et al. 1998). Other analyses based on reports of treatment facilities to State administrative data systems have found that in 2002, criminal justice/DWI referrals accounted for 40 percent of admissions for treatment of alcoholism only and for 34 percent of admissions for treatment of abuse of alcohol and another drug (SAMHSA 2004). Referrals of impaired drivers make up the vast majority of justice system referrals to the public treatment system (i.e., treatment programs that are not located in jails or prisons).

## Screening in the Criminal Justice System

### Definition and Purpose of Screening

The main goal of screening criminal justice offenders is to identify those likely to have alcohol (or drug) use disorders. Another purpose of screening in some jurisdictions is to identify those who may benefit from sanctions, such as house arrest or electronic monitoring, that may restrict AOD use and reduce recidivism of individual offenders. In medical settings, short questionnaires can be used to screen people for alcohol use. In the criminal justice system, however, screening often incorporates procedures usually considered part of a more comprehensive assessment, such as more in-depth interviews, because offenders may be motivated to under-report their alcohol-related problems (see the section “Limitations of Current Screening Procedures”). Assessment in the criminal justice system typically involves examining the severity of a person’s AOD or mental health problems; this assessment then guides the development of a treatment plan. Together, screening and assessment aim to determine the need for further assessment, to ascertain which offenders need specialized treatment services, to match offenders’ treatment needs to appropriate services, and to determine the appropriate placement of offenders within different institutional units or facilities (Peters and Bartoi 1997).

### Current Practices for Screening Offenders

#### Non-DWI Offenders

There are no nationwide standards for whether or how non-DWI offenders should be screened for alcohol use. Similarly, the literature is largely silent on alcohol screening and brief interventions for people convicted of crimes other than DWI. Some drug court programs include screening components, but these have been neither well described nor well studied. Therefore, the remainder of this article mainly discusses the published literature that specifically addresses screening of DWI offenders.

#### DWI Offenders

Most States mandate screening to evaluate the alcohol abuse problems of DWI offenders and to determine the offenders’ needs for further assessment and treatment (Chang et al. 2002). Current guidelines for sentencing DWI offenders recommend that all offenders should be screened for alcohol and drug use problems and recidivism risk (NHTSA and NIAAA 1996), but the existing screening programs for DWI offenders differ in how they evaluate clients. Some programs conduct a simple screening—typically, a brief questionnaire—to determine whether the client should be transferred either to an education program or to treatment. Other programs combine screening with assessment and provide referral guidelines and specific treatment recommendations. These programs typically comprise three activities (Chang et al. 2002):

- Testing—the use of self-report instruments (i.e., questionnaires) to evaluate the offender’s AOD use and related problems.
- Interviewing/assessment—face-to-face interactions between specially trained personnel and the offender to review the offender’s test results, further clarify the circumstances of the arrest, and identify family, medical, personal, or legal problems that may indicate a need for treatment.
- Referral and monitoring—referral of offenders for appropriate services, tracking their progress through the system, and assessing their compliance with court-mandated treatment.

Ninety percent of screening programs surveyed report that they use both in-person interviews and self-report questionnaires (Chang et al. 2002). The most commonly used standardized instruments in DWI screening programs are the Mortimer-Filkins (MF) test (Wendling and Kolody 1982), the Michigan Alcoholism Screening Test (MAST) (Selzer et al. 1971), and the Driver Risk Inventory (DRI).^2^

## Special Considerations Regarding Screening in Criminal Justice Populations

### Limitations of Current Screening Procedures

One factor limiting the effectiveness of current screening procedures may be the use of screening instruments that are not designed to evaluate offenders. Most instruments, such as the commonly used MAST, were developed in populations other than DWI offenders or other criminal justice populations and were not designed specifically for use in court-mandated screening (Chang et al. 2002). Furthermore, it has been suggested that screening in the criminal justice system should move beyond alcohol-specific measures to include misuse of other drugs and psychosocial factors that often coexist with alcohol abuse and dependence. For example, screening procedures should be able to reliably determine symptoms of other drug use and misuse, history of violent behavior, motivational factors, lifestyle factors, medical history, and psychiatric problems (Peters and Bartoi 1997). To date, no available instrument has demonstrated accuracy to screen for both psychiatric problems and AOD misuse. Therefore, it may be useful to develop specialized mental health and AOD abuse screening instruments for evaluating criminal justice clients (Peters and Bartoi 1997).

Another potential limitation of current screening procedures is that all standardized instruments currently used for evaluating DWI offenders rely almost exclusively on self-reported information. Many offenders under-report their drinking, however, either unconsciously or because they want to avoid being labeled as having alcohol problems. Therefore, screening based on self-reports may underestimate the number of clients in need of intervention.

### The Coercive Nature of Court-Ordered Screening

Prompt and appropriate intervention after a DWI or other alcohol-related offense might offer offenders a unique opportunity to enter treatment without having to seek it on their own. Some offenders accept the need for screening and treatment, and several studies have demonstrated that clients who were ordered into alcoholism treatment by the criminal justice system showed reductions in alcohol use and illegal activities similar to clients who had entered treatment voluntarily (Hubbard et al. 2002; Summers 2002). Other offenders, however, feel coerced into screening and treatment and resist the process, or may fear that if they report having alcohol use problems they may be penalized by receiving unfavorable custody assignments or probation conditions (Knight et al. 2002). Finally, offenders may deny or minimize their alcohol problems to avoid the costs of court-ordered treatment (Chang and Lapham 1996). All of these factors can make it difficult to ascertain the true nature and severity of an offender’s substance use problems (Chang et al. 2001), and they underscore the need for adequately trained personnel to conduct screening in criminal justice populations to detect any under-reporting. Well-trained interviewers may be more adept at developing rapport with clients and eliciting more accurate responses. Many programs, however, cannot afford specially trained staff for these evaluations (Knight et al. 2002).

### Underdiagnosis of Alcohol-Related Problems

As a result of the limitations of current screening procedures and the coercive nature of court-ordered screening, offenders’ alcohol-related problems often are underdiagnosed. This is illustrated by a study that determined the rates of alcohol abuse and dependence in a population of 1,078 convicted DWI offenders (Lapham et al. 2004). Diagnoses for these offenders were based on two sets of data—information obtained during an initial, court-ordered screening, and self-reports obtained during a voluntary, noncoerced interview 5 years after the participants were initially screened. The initial screenings employed master’s degree–level evaluators and involved extensive testing, including onsite breath alcohol testing, as well as input from friends or relatives of the offenders. Five years later, a standardized diagnostic interview (the Diagnostic Interview Schedule) (Robins et al. 1981) was used to ascertain self-reported symptoms of alcohol use disorders and age of onset. The investigators found that at the initial screening, 17 percent of the offenders reported alcohol consumption patterns consistent with alcohol abuse, and 20 percent reported patterns consistent with alcohol dependence. At the interview conducted 5 years later, however, 20 percent reported symptoms of alcohol abuse, and 60 percent reported symptoms of alcohol dependence that had already begun when they were initially screened. These findings demonstrate that coerced screening in the criminal justice system may not correctly identify all offenders in need of further interventions.

### Timing of the Screening and Intervention

Offenders may be screened at various stages of the judicial process, including at arrest or arraignment, at pretrial investigation, during interactions with court staff, or as a postsentence action. Just as laws are most likely to deter illegal behavior (e.g., DWI) if they are perceived to result in swift, certain, and severe sanctions (e.g., Morral et al. 2002), screening and interventions with offenders who have alcohol use disorders probably will be more effective if they are initiated soon after the offense. These situations can be equated to the “teachable moments” observed in primary care or emergency medical settings—situations in which a patient may be particularly amenable to an alcohol intervention (e.g., when receiving acute medical care for an alcohol-related injury).^3^ The process of adjudicating offenders is long, however, often spanning months or even years. In many instances, before screening can be initiated, cases first must be scheduled, pleas entered, and—if an offender pleads not guilty—the case must go to trial, which may take 6 months or more after the offense. Even if an offender is convicted, more time may pass before a hearing is set for sentencing (which may include a requirement to undergo screening or begin treatment). Such delays can postpone the initiation of treatment and supervision. By the time the offender is referred for screening and/or treatment, the “teachable moment” may have passed.

### Financial Constraints

With the tight budgets in most communities and States, criminal justice systems faced with increasing numbers of incarcerated and nonincarcerated offenders and probationers are experiencing severe financial constraints. As a result, criminal justice systems are seeking to transfer much of the costs for alcohol screening and intervention to the offenders, particularly those who do not receive jail sentences for their offenses. For example, a survey of court processes for DWI screening revealed that only four States did not charge non-incarcerated offenders a fee for screening (Chang et al. 2002). Most DWI programs are supported by clients, who pay 100 percent of fees. In addition, offenders often are burdened by court costs, fines, attorney fees, and missed work time, and they may have to pay for their own treatment. The prospect of having to pay for screening as well as treatment may increase offenders’ motivation to avoid a treatment referral by under-reporting their alcohol consumption. Similarly, programs that receive referrals from the criminal justice system may have limited financial resources, and applying for reimbursement from insurance providers can involve high administrative expenses with no guarantee of payment. These factors may be powerful incentives for both offenders and treatment programs to under-identify alcohol use disorders (Woody and Forman 2001).

Financial constraints also affect the screening, assessment, and treatment of incarcerated offenders. Few correctional agencies have the financial resources to provide comprehensive assessment for all newly admitted inmates (Knight et al. 2002). Lack of financial means also may limit the provision of treatment, even though other analyses have shown that, despite variations in treatment costs among various programs, in most cases these costs are considerably lower than the costs of incarceration in prisons or jails (Vigdal 1995).

### Confidentiality Concerns

Federal regulations that serve to protect the confidentiality of patients receiving AOD abuse therapy also apply to criminal justice clients. Restrictions to prevent disclosure of information that would identify an offender as an alcohol abuser govern issues such as whether and how program staff may contact sources of information (e.g., families, employers, and other service providers) or how the agencies responsible for the offender’s welfare communicate with each other about the offender’s assessment or treatment progress. Particularly when a team approach involving several agencies is used to screen and treat criminal justice clients, these confidentiality regulations can interfere with effective intervention.

Information protected by Federal confidentiality regulations may be disclosed if the offender has signed a proper consent form. Obtaining the offender’s voluntary consent to information disclosure is the most commonly used method for allowing communication between the staff members of different agencies collaborating in the adjudication and care of AOD-abusing offenders (Vigdal 1995).

### Comorbidity

Research indicates that many offenders in the criminal justice system not only have alcohol use disorders but are likely to have other drug-related problems and mental illnesses as well. At least one-quarter of alcohol-dependent offenders have lifetime histories of major depression or some form of bipolar disorder. One study revealed that 44 percent of jailed inmates in a metropolitan jail had lifetime substance use disorders concomitant with either depression or antisocial personality disorder (Vigdal 1995). High rates of comorbidity were confirmed in a study of first-time DWI offenders who were interviewed 5 years after being referred to screening following their DWI offense (Lapham et al. 2001). This study found that:

- 85 percent of the female and 91 percent of the male DWI offenders studied had met the criteria for an alcohol use disorder (i.e., abuse or dependence) at some time in their lives.
- 32 percent of the female and 38 percent of the male offenders had met the criteria for abuse of or dependence on another drug at some time in their lives.
- Among the offenders with an alcohol use disorder, 50 percent of the women and 33 percent of the men also had at least one other psychiatric disorder (other than abuse of or dependence on another drug).
- The most commonly occurring comorbid disorders were depression and post-traumatic stress disorder.

These findings indicate that criminal justice populations, including DWI offenders, should be evaluated for psychiatric problems commonly co-occurring with alcohol use disorders. This is especially important because studies in other populations have shown that alcohol-dependent patients with coexisting psychiatric disorders have worse treatment outcomes than patients without comorbid disorders (Ciraulo et al. 2003; Compton et al. 2003).

### Screening as Brief Intervention

In various medical settings, brief interventions are recommended for patients who misuse alcohol and are at risk for dependence, but who are not alcohol dependent. These interventions typically:

- Involve four or fewer sessions
- Are conducted in a nontreatment setting (i.e., not in a specialized alcoholism treatment facility), and
- Are performed by health care providers and others who are not specialized in addiction treatment.

One advantage of brief interventions is that they can be administered at a relatively low cost. For example, Zarkin and colleagues (2003) found that the costs of screening and brief interventions in a managed care setting were only a few dollars per client. However, additional studies are needed to determine the exact costs and benefits of screening and brief interventions in criminal justice populations.

The process of screening DWI offenders for alcohol use disorders shares several of the characteristics of brief interventions. For example, screening usually involves one or two visits with the offenders. Therefore, screening itself is likely to have some impact on offenders’ drinking behavior. Consistent with this assumption, practitioners have recognized for more than 15 years that simply asking people about their drinking and its consequences can positively affect those people’s drinking patterns (Institute of Medicine 1990). Consequently, it appears plausible that current screening procedures could be redesigned as brief interventions to help offenders develop insight into how alcohol affects their lives and to motivate them to confront the problem. In some cases, screening and brief intervention may reduce the need for more intensive treatment. In other cases, this approach might motivate offenders to follow through with recommended treatment interventions. To date, however, the effectiveness of the screening process in reducing alcohol use or recidivism among offenders has not been evaluated.

**At a Glance**

**Screening in the Criminal Justice System**

In 2002, 40 percent of admissions to alcoholism treatment alone, and 34 percent of admissions to treatment programs for abuse of alcohol and other drugs, were accounted for by criminal justice/DWI referrals (SAMHSA 2004).

Court-ordered screening misses many people with alcohol use disorders. In a study of 1,078 convicted offenders court-ordered to be screened for alcohol problems, lower proportions reported alcohol consumption patterns consistent with alcohol abuse or alcohol dependence at the initial screening than at a later voluntary screening (Lapham et al. 2004).

Limitations of screening procedures in the criminal justice system include:

- No screening instruments are available that have proven validity to assess both AOD use and the range of mental health problems found in criminal justice populations.
- No screening instruments are available specifically for criminal justice offenders.
- Current screening instruments rely almost exclusively on self report.
- Court-ordered screening is by definition coercive.
- Screening and treatment programs have limited financial resources; costs may be passed on to people being screened or treated who may be unable to pay.

## Brief Interventions in the Criminal Justice System

The appropriateness and efficacy of using brief interventions for offenders with AOD use disorders is undergoing evaluation. Such interventions, which typically consist of one to four treatment sessions and therefore are much shorter than traditional alcoholism treatment approaches, are increasingly being used in a variety of settings for clients with alcohol problems. Numerous types of brief interventions have been developed, ranging from advising clients to cut down on or quit drinking, to brief screening and feedback on results, motivational interventions, and contingency contracting. (For a review of such interventions, see Poikolainen 1999, NIAAA 1999, and Babor and Higgins-Biddle 2001.) The effectiveness of brief interventions in reducing alcohol consumption among both alcohol abusers and those with alcohol dependence has been demonstrated in a variety of settings (e.g., see Moyer et al. 2002; also see the article by Moyer and Finney in the companion issue). However, few studies have evaluated the impact of brief interventions in criminal justice populations. Two studies that have been conducted with groups of DWI offenders are described in the next section. Both of these analyses used motivational approaches.

### Brief Motivational Interventions for DWI Offenders

Brief motivational interventions for alcohol and drug misuse increasingly are being introduced into the criminal justice system, and their effectiveness now is being evaluated. Davis and colleagues (2003) examined the efficacy of brief motivational feedback in increasing treatment participation of offenders with substance use disorders following completion of their jail sentences. The investigators found that offenders receiving feedback were more likely to schedule appointments for followup treatment than were control group offenders.

A study conducted among first-time DWI offenders attending a court-mandated intervention assessed the effects of adding two 20-minute individual sessions and a brief followup session to a traditional intervention, which consisted of a drinking assessment plus three 2½-hour sessions of group discussion and exercises (Wells-Parker and Williams 2002). In this study, the addition of the brief interventions reduced recidivism only among offenders with evidence of depression, but not among nondepressed offenders. This finding suggests that brief interventions may be particularly useful in certain subgroups of DWI offenders. Ongoing studies should further clarify the effects of brief interventions in reducing recidivism among convicted DWI offenders.

### Availability and Effectiveness of Treatment

Offenders identified during screening as having a high probability of being alcohol dependent ideally should be referred for further assessment and treatment. However, referral decisions must consider the availability and accessibility of treatment services during and after incarceration (Knight et al. 2002). For example, many (if not most) offenders have no health insurance. The Arrestee Drug Abuse Monitoring survey (National Institute of Justice 2003) found that in 2000, the proportion of adult arrestees at risk for drug dependence who had the health insurance coverage needed to address the problem was relatively low. In half of the sites sampled, at least two-thirds of those at risk lacked any type of health insurance. Thus, even if treatment is available to these offenders, it may not be accessible because of financial concerns. This places the burden for paying for treatment services largely on the public sector.

Furthermore, the need for treatment services greatly exceeds the supply, especially in rural areas of the United States. As a result, offenders routinely are mandated by the courts to attend Alcoholics Anonymous meetings, either as their primary treatment or in combination with or after the completion of other treatment programs. However, the effectiveness of the AA approach for criminal justice populations has not been determined.

Another promising approach for treating alcohol abusers is the use of medications that reduce craving for alcohol (e.g., naltrexone). These medications have been shown to be effective in preventing relapse when used in combination with psychosocial treatment such as brief motivational interventions. These pharmacotherapies are expensive, however, and their effectiveness has not been evaluated in criminal justice populations. Furthermore, compliance with taking the medications as prescribed may be low in these populations.

### Summary

In summary, not much is known about the effectiveness of various treatment approaches, particularly brief interventions, in criminal justice populations, including DWI offenders. Research conducted in other settings indicates that brief interventions can help patients reduce alcohol consumption and adverse consequences. Although these approaches should be effective with significant numbers of criminal justice clients, more studies are needed to establish their effectiveness with this group.

A variety of other strategies that have proven useful for treating alcohol problems in the general population also may be appropriate for convicted DWI offenders. Because a significant proportion of these offenders meet diagnostic criteria for alcohol abuse or dependence, strategies found successful with people seeking treatment for their alcohol problem are likely to prove successful with significant segments of the DWI population. Therefore, alcoholism treatment for DWI populations can have a positive effect on public safety by reducing recidivism, as well as on public health by reducing the negative health and social consequences associated with excessive drinking.

## Future Outlook

Alcohol abuse and dependence are highly prevalent among offenders in the criminal justice system, particularly among DWI offenders. For many of these people, screening and intervention could offer a valuable opportunity to reduce alcohol use and break the cycle of alcohol misuse and resulting criminal activities. However, a number of factors limit offenders’ access to comprehensive screening and treatment. In addition, the effectiveness of screening and the potential therapeutic effects of screening and brief intervention in these populations have not yet been evaluated adequately. Conducting this research is a challenge (Belenko 2002). Few judges will agree to alter court proceedings so offenders can be randomly assigned to different treatment or evaluation conditions. Moreover, it is inappropriate to deny existing services to selected offenders in order to establish a control group. As a result, few randomized controlled studies of screening and brief intervention have been conducted successfully in partnership with court systems (Belenko 2002).

So how can the availability of screening and brief intervention in the criminal justice system as well as their effectiveness in reducing recidivism be improved? Several options are possible:

- The procedures used to screen people entering the criminal justice system should be reviewed and retooled to better address the range of alcohol use and mental health problems found in criminal justice populations.
- More rigorous research studies should evaluate the effectiveness of screening and brief interventions for reducing recidivism among offenders. The current lack of evidence is particularly problematic considering the large number of people arrested each year for alcohol-related offenses and their high recidivism rates.
- More emphasis should be placed on understanding how different groups of alcohol-dependent offenders (e.g., women with post-traumatic stress disorder or young men with antisocial personality disorder) fare within different screening and treatment modalities. When possible, research studies should include equivalent comparison groups.

Despite the limitations of the existing research, investigators have gained sufficient knowledge regarding screening and brief interventions in the criminal justice system to recommend the following improvements to existing programs:

- A national strategy should be developed to improve and standardize current screening systems.
- Standards should be established for training and qualifying personnel conducting screening.
- Techniques of brief motivational interventions and similar evidence-based intervention approaches should be evaluated in criminal justice settings and incorporated into screening protocols. Given their effectiveness in other populations, it would be appropriate to include these approaches in education programs designed for DWI offenders who, based on screening results, are not alcohol dependent.
- Education programs designed for DWI offenders should include ongoing assessment for alcohol use disorders that may not have been identified during the initial screening or assessment. This is particularly important because alcohol-related problems often are underdiagnosed in these offenders.
- Less emphasis should be placed on self-reports and more emphasis on externally validated information (i.e., examination of court records for previous alcohol-related offenses, use of monitoring devices, and use of collateral information [e.g., from the offender’s family or others]) before making recommendations regarding possible interventions.
- Screening should address other drug use and mental health disorders that frequently co-occur with alcohol use disorders.
- Treatment services should be made accessible and affordable.

Although these measures are associated with additional expense, the costs to society of failing to properly evaluate and treat alcohol abusers in the criminal justice system also are great. Tax dollars support law enforcement activities, the judicial system, and the costs of building and staffing jails and prisons. In contrast, appropriately delivered treatment costs much less than incarceration and can effectively change behavior and reduce re-arrests (Vigdal 1995). Thus, developing programs to improve screening and add cost-effective brief interventions to the existing screening and treatment processes holds great promise for rehabilitating offenders with alcohol-related problems.

## Footnotes

1 The term “alcohol misuse” refers to any type of alcohol use that has harmful consequences (e.g., a single case of intoxication leading to an alcohol-related traffic crash), regardless of whether it meets the diagnostic criteria for alcohol abuse or alcohol dependence.

2 The Driver Risk Inventory (DRI), developed in 1987, is available from Behavior Data Systems, P.O. Box 32308, Phoenix, AZ 85064.

3 It should be noted, however, that many alcoholics in the immediate postintoxication period suffer from neurocognitive deficits that may impair their ability to learn new information. As a result, interventions delivered during these “teachable moments” may not be as effective.
